# From site-level to regional adaptation planning for tropical commodities: cocoa in West Africa

**DOI:** 10.1007/s11027-016-9707-y

**Published:** 2016-03-10

**Authors:** Götz Schroth, Peter Läderach, Armando Isaac Martinez-Valle, Christian Bunn

**Affiliations:** 1 C.P. 513, 68109-971 Santarém, Pará Brazil; 2 International Center for Tropical Agriculture (CIAT), Managua, Nicaragua; 3 Department of Agricultural Economics, Humboldt University, 10115 Berlin, Germany

**Keywords:** Adaptation planning, Climate change vulnerability, Crop change, Diversification, Intensification, *Theobroma cacao*, Zoning

## Abstract

The production of tropical agricultural commodities, such as cocoa (*Theobroma cacao*) and coffee (*Coffea* spp.), the countries and communities engaged in it, and the industries dependent on these commodities, are vulnerable to climate change. This is especially so where a large percentage of the global supply is grown in a single geographical region. Fortunately, there is often considerable spatial heterogeneity in the vulnerability to climate change within affected regions, implying that local production losses could be compensated through intensification and expansion of production elsewhere. However, this requires that site-level actions are integrated into a regional approach to climate change adaptation. We discuss here such a regional approach for cocoa in West Africa, where 70 % of global cocoa supply originates. On the basis of a statistical model of relative climatic suitability calibrated on West African cocoa farming areas and average climate projections for the 2030s and 2050s of, respectively, 15 and 19 Global Circulation Models, we divide the region into three adaptation zones: (i) a little affected zone permitting intensification and/or expansion of cocoa farming; (ii) a moderately affected zone requiring diversification and agronomic adjustments of farming practices; and (iii) a severely affected zone with need for progressive crop change. We argue that for tropical agricultural commodities, larger-scale adaptation planning that attempts to balance production trends across countries and regions could help reduce negative impacts of climate change on regional economies and global commodity supplies, despite the institutional challenges that this integration may pose.

## Introduction

A number of studies have highlighted the vulnerability to climate change of agricultural commodities in key producing regions, emphasizing the fact that climate change poses threats not only to individual farmers and communities, but also to the economies of affected regions, the global supply of the respective commodities, and the sustainability of the industries concerned (Hannah et al. 2013; Bunn et al. 2015). In other words, climate change adaptation is not only the local process as which it is often seen (Agrawal 2008; Pelling 2011), but is a regional and global need for the respective economic sectors. Among the better-studied commodities under threat from climate change is Arabica coffee (*Coffea arabica*). For this crop, several global and regional studies have shown threats to quantity and quality of production from projected future temperature increase, rainfall decrease, increased frequency of extreme weather events, and associated changes in pest and disease pressures (Jaramillo et al. 2009; Baca et al. 2014; Ovalle-Rivera et al. 2015). In Central America, for example, globally significant Arabica coffee production coincides with projections of severe climate changes and this has attracted attention from international development agencies and the industry.

Cocoa (*Theobroma cacao*) in West Africa, on the other hand, has received less attention, despite the fact that 70 % of global cocoa production is concentrated here. Such an extreme concentration of the production of a commodity in one geographical region makes the global industry highly vulnerable to a regional decline in climatic suitability. Cocoa is an understory tree of the Amazon forest, and as such it is sensitive to prolonged drought and high dry season temperatures. Compared to other cocoa producing regions, West Africa’s cocoa belt is already exposed to a considerable drought risk (Wood and Lass 2001; Ruf et al. 2015) and is now projected to become increasingly affected by maximum temperatures approaching the crop’s physiological tolerance limit (Läderach et al. 2013; Schroth et al. 2015b). This situation is further compounded by the spread of low or zero-shade production practices in much of West Africa, progressively replacing the traditional practice of growing cocoa under the shade of remnant forest trees (Ruf 2011). This high vulnerability to climate change risks to affect the economies of several West African countries for which cocoa is among the most important agricultural exports (International Trade Centre 2001), as well as the livelihoods of several million cocoa farmers and their families.

Projections of national or regional decline in climatic suitability can, however, easily obscure an often pronounced small-scale heterogeneity in climatic conditions and vulnerabilities within the same country or region (Baca et al. 2014). As a result of this heterogeneity, the projected impacts on climatic suitability for a given crop may be strongly negative in some parts but much less negative or even positive in other parts of the same country or region. For example, a recent study of Arabica coffee in Indonesia suggested that while the climatic suitability of some islands for the crop may strongly decline over the next decades, conditions on other islands may even permit a future expansion of coffee production (Schroth et al. 2015a). This situation calls for an integrated national or even regional approach to climate change adaptation that not only attempts to minimize hardships to farmers and communities in the most affected areas, but also identifies areas where climate projections would allow the production of key commodities to be sustained or increased. Within such a regional strategy, site-level recommendations could then range from support to farmers to gradually transition to other crops in the most affected areas (Eyshi Rezaei et al. 2015), to adjustments in farming practices without the need for changing main crops in areas of intermediate climate impact (Waha et al. 2013), to specific programs and policies of intensification and expansion of production in areas where climate projections are favorable. The overall impact of climate change on national and regional economies and global commodity supplies could thus be minimized.

In practice, however, dividing a region like West Africa into adaptation zones where coordinated but differentiated adaptation actions could be implemented has to take into account several complicating factors. For one, climate variables such as temperature and rainfall may show different trajectories, requiring integrated measures of climate suitability. Second, climate change impacts depend on the time horizon considered; therefore boundaries between impact zones will shift over time and may be arbitrary. A third question is how to handle the uncertainty of climate change projections as seen in the variation among global circulation models (GCMs) (IPCC 2013), which is relatively high for West Africa (Jalloh et al. 2013; Niang et al. 2014).

Our objective here is to illustrate a regional approach to climate change adaptation for tropical agricultural commodities by applying it to cocoa in West Africa where recent studies have indicated overall negative but spatially highly differentiated climate change impacts (Läderach et al. 2013). While our analysis and recommendations are specific to cocoa, the concept and methodology are relevant and applicable to other crops as well. Our approach is based on the relative current and future climatic suitability for cocoa farming as simulated by the ecological niche model Maxent. We first justify our choice of the average of available global circulation model (GCM) projections as the basis for developing a regional adaptation strategy by contrasting it with two alternative courses of action. We then focus on a practical approach to converting those results into a spatially differentiated but regionally integrated adaptation strategy that takes into account both risks and opportunities for the future development of cocoa farming. We see our approach as adaptive in nature and requiring periodic updates to ensure that new information is continuously fed into the decision-making process, as should be the case with all adaptation planning and implementation. We also emphasize the need to complement our top down planning approach through a bottom up engagement with local stakeholders to specify locally appropriate adaptation measures. We see our approach as a contribution towards a greater institutionalization of the adaptation process moving climate change adaptation from local crisis response to a regional process of flexible and adaptive planning of sustainable rural development under climate change conditions.

## Methods

### Modeling current and future climatic suitability of cocoa in West Africa

We characterized the current climate of the West African cocoa belt by creating a map of the current extent of cocoa farming in the area and overlaying it with climate variables from the WorldClim database (www.worldclim.org; Hijmans et al. 2005). For the purpose of this study, we defined the West African cocoa belt as the cocoa producing areas between Sierra Leone in the west and Cameroon in the east (International Trade Centre 2001). For the extent of cocoa farming in this area we used a map from the Atlas on Regional Integration in West Africa (ECOWAS 2007) as a basis, except for Nigeria where we used a map of cocoa producing districts from the 2007 national cocoa production survey (CRIN 2008). We updated these maps with literature and field information. Specifically, we included all of Liberia as cocoa producing area because a recent report shows some cocoa production for essentially every part of the country (CAAS 2007; Schroth et al. 2015b). We also included into the cocoa area the wet, southwestern parts of Côte d’Ivoire and Ghana where cocoa farming has expanded relatively recently (Ruf et al. 2015). The resulting, combined polygon reflects the minimum area where environmental conditions, including climate, have been sufficiently suitable for cocoa farming in recent years for local farmers to have opted for cocoa rather than other crops available to them. From the cocoa production area we then sampled 558 evenly spaced points on a 0.3 degree grid that were used for the calibration of our Maxent model of relative climatic suitability, as explained below.

The WorldClim data were generated through interpolation of average monthly climate data from a global network of 47,554 meteorological stations on a 30 arc-second resolution grid often referred to as 1 km resolution. Only stations for which there were more than 10 years of data were included, calculating means of the 1950–2000 period referred to here as current or present climate. WorldClim includes data from 751 climate stations for the West African cocoa belt. Of these, 657 stations have precipitation data, 442 stations have mean temperature data, and 120 stations have data on temperature extremes. The database lists values for derived, bioclimatic variables that are often used in ecological niche modeling. These represent averages (e.g., mean annual temperature and precipitation), seasonality (e.g., annual range in temperature and precipitation) and extreme environmental factors (e.g., temperature of the coldest and warmest month, precipitation of the wettest and driest quarters; Appendix Table 4). To these bioclimatic variables provided by WorldClim, we added a set of variables that were specifically intended to reflect the sensitivity of cocoa to drought (Wood and Lass 2001; Carr and Lockwood 2011). From the WorldClim information, we calculated for each location the number of consecutive months with less than 100 mm of rainfall which is often used to characterize the length of the dry season for cocoa (Wood and Lass 2001). Furthermore, following the approach taken by Läderach et al. (2013) for modeling climate vulnerability of cocoa in Côte d’Ivoire and Ghana, we added eight variables intended to reflect the response of potential evapotranspiration (ETP) to temperature variation. We estimated ETP with the Hargreaves equation (Hargreaves and Samani 1985):

$$ \mathrm{E}\mathrm{T}\mathrm{P} = 0.0023\kern0.1em \cdotp \kern0.1em \mathrm{R}\mathrm{a}\ \cdotp \kern0.1em {\left(T-t\right)}^{0.5}\kern0.1em \cdotp\ \left(\mathrm{t}\mathrm{m} + 17.8\right)\mathrm{mm}\ {\mathrm{day}}^{\hbox{-} 1} $$

where ETP is evapotranspiration in mm per day; Ra is extraterrestrial solar radiation expressed as water equivalent in mm per day; *(T* − *t)* is the difference between monthly mean maximum and mean minimum temperature in degrees centigrade; and tm is the mean air temperature in degrees centigrade. The Hargreaves method requires less data than the well-known Penman-Monteith method while the results of the two methods are closely correlated (Hargreaves and Allen 2003). Solar radiation was estimated for each month using the “shortwavc.aml” algorithm (Kumar et al. 1997) which requires as input a Digital Elevation Model (DEM) (Reuter et al. 2007), the location and the time period. The output is given in kJ m^−2^ month^−1^, which we converted into water equivalents in mm day^−1^, considering that 1 mm day^−1^ = 2.45 MJ m^−2^ day^−1^ (Allen et al. 1998). From this we calculated monthly ETP values. We applied the same concept of annual trends and extreme or limiting environmental factors as for the temperature and precipitation related bioclimatic variables to calculate eight additional ETP variables (Appendix Table 4).

For the future climates, we used from the 34 global circulation models (GCMs) included in the IPCC Fifth Assessment Report (2013) projections of 15 GCMs for the 2030s period (average of 2020 to 2049) and 19 GCMs for the 2050s period (average of 2040 to 2069) for which projected climate data had the necessary spatial resolution (Appendix Table 5). To increase the spatial resolution of the GCM results, which is in most cases more than 100 × 100 km and therefore inappropriate for analyzing the impacts on agriculture, we used a statistical downscaling method (named delta method), based on the sum of interpolated anomalies to high-resolution monthly climate surfaces from WorldClim (Hijmans et al. 2005). The method produces a smoothed (interpolated) surface of changes in climates (deltas or anomalies) and then applies this interpolated surface to the baseline climate, taking into account the possible bias due to the difference in baselines. The method assumes that changes in climates are only relevant at coarse scales, and that relationships among variables are maintained towards the future (Ramirez-Villegas and Jarvis 2010). We downloaded the data from the Climate Change and Food Security (CCAFS) Program’s GCM portal (http://www.ccafs-climate.org/) and applied the downscaling method on the 15 or 19 GCMs, respectively, for the intermediate emission scenario RCP 6.0 (Moss et al. 2010; Van Vuuren et al. 2011).

To characterize the relative suitability for cocoa of the current and projected future climate distribution within the cocoa belt, we developed a spatial, statistical model of current and future climatic suitability for cocoa in the West African cocoa belt that integrates a large number of climate variables. For this task we used a statistical niche model, Maximum entropy (Maxent), that incorporates crop-environment interactions through a machine learning approach based on the current climatic conditions in cocoa growing areas (Phillips and Dudik 2008). Maxent is a general-purpose method for making predictions or inferences from incomplete information. Similar to logistic regression, Maxent weighs each environmental variable by a constant. The probability distribution is the sum of each weighted variable divided by a scaling constant to ensure that the probability value ranges from 0 to 1. The algorithm starts with a uniform probability distribution and iteratively alters one weight at a time to maximize the likelihood of reaching the optimum probability distribution. Maxent is generally considered to be among the most accurate models for this task (Elith and Graham 2009). Its ability to predict species occurrence probabilities has been shown to be statistically better than that of alternative models such as CaNaSTA, Domain, and Bioclim (Läderach et al. 2009). This approach has previously been used to model relative climatic suitability of cocoa in West Africa (Läderach et al. 2013) and for other tree crops including coffee elsewhere (Schroth et al. 2009, 2015a; Baca et al. 2014).

Climatic suitability for cocoa in the context of this analysis refers to the probability (in percent) that cocoa can be profitably farmed at a site, judged from the combined presence of climatic conditions that characterize other known sites of current cocoa cultivation. Not all areas identified by Maxent as climatically suitable actually grow cocoa since some may have unsuitable soil or be occupied by human settlements, protected areas or different crops. For calibrating the climate model, we used the 558 sampling points that had been generated by systematically sampling the cocoa production areas in the cocoa belt at a 0.3 degree grid, as explained before. In addition, a random background (“pseudo absence”) sample at a 5:1 ratio of background to calibration points was drawn from the area of the countries of the cocoa belt excluding points of known cocoa presence (for a discussion of sample to background ratios see Barbet-Massin et al. (2012)). The climatic conditions at the calibration points of known occurrence and random pseudo absence of cocoa according to the climate surfaces created from the WorldClim data were used to train the Maxent algorithm and estimate the spatial distribution of relative climatic suitability for cocoa.

We initially ran the Maxent model with the full set of variables listed in Appendix Table 4, then further optimized the model by reducing the weight of climate variables that vary across the region and have a significant influence on the model result, but are not of critical importance for the suitability of an area for growing cocoa. We did this in an iterative way. We started with model runs including all climate variables in Table 4 and identified for each run those variables that explained more than 5 % of the variability in the model result. For each of these variables, we then made an expert judgment whether or not it was meaningful as a differentiating factor of climatic suitability for cocoa for this specific area. Variables making an important contribution to the model outcome that we considered agro-ecologically not meaningful for this specific situation were eliminated and the model was re-run. This process resulted in the exclusion from the initial model of the variables for ETP of the wettest month (ETP2), ETP of the wettest quarter (ETP4), precipitation of the coldest quarter (BIO 19), and ETP of the coldest quarter (ETP 7) on the grounds that in a humid tropical forest area during the wet season (which includes the coldest quarter) water supply is normally abundant and a difference in rainfall or ETP would not be expected to make a critical difference for the crop. With this approach we attempted to combine the advantages of two common approaches to variable selection for species distribution models: (i) the use of a large number of pre-selected variables, and (ii) the use of expert knowledge in variable selection (Porfirio et al. 2014). Test model runs showed that excluding these four variables slightly reduced the severity of the projected climate change impacts. In the final model, variables related to dry season rainfall and maximum temperatures during the dry season made the largest contributions to explaining variation in current climatic suitability for cocoa (Appendix Table 6). We applied this model to the maps of climate data to estimate the spatial distribution of relative climatic suitability for cocoa for current, 2030s and 2050s climatic conditions. The model was run with Maxent’s own software. Model performance was verified by computing the area under the receiver operating characteristic curve (AUC) as a measure of model skill (Peterson et al. 2008). Using the 558 evidence points, 20 model runs were performed, using 80 % of the points for model training and the remaining 20 % for model testing. The performance was high with an AUC value of 0.976 on the test data on average of the 20 model runs on a scale from 0.5 for a chance model to 1 for a perfect model (Peterson et al. 2008). The results were analyzed and maps created in ArcGIS 10.1, and statistics were estimated using R.

### Zoning approach

We characterized the variability among the 15 (for 2030s climate) or 19 (for 2050s climate) GCMs, respectively, for key climate variables by computing the average, lowest quartile and highest quartile of these variables for the various GCMs. Values for each variable were then averaged over the entire cocoa belt (Appendix Table 4).

To characterize the effect of the uncertainty in climate projections, we applied the Maxent model for each GCM separately and then calculated the average, lowest quartile and highest quartile of the 15 or 19 model outputs, respectively. This resulted in a time sequence of climate suitability distributions (current, 2030s and 2050s climates) for the most likely case (average of all GCMs), a pessimistic case (lowest quartile of GCMs), and an optimistic case (highest quartile of GCMs). We used these different projections to discuss the feasibility of basing an adaptation strategy on either the range of reasonably likely climate change scenarios (“all-encompassing strategy”); (ii) the pessimistic projections of the lowest quartile (“safety-first strategy”); and (iii) the average of all GCMs (“most likely scenario strategy”).

**Fig. 1:**
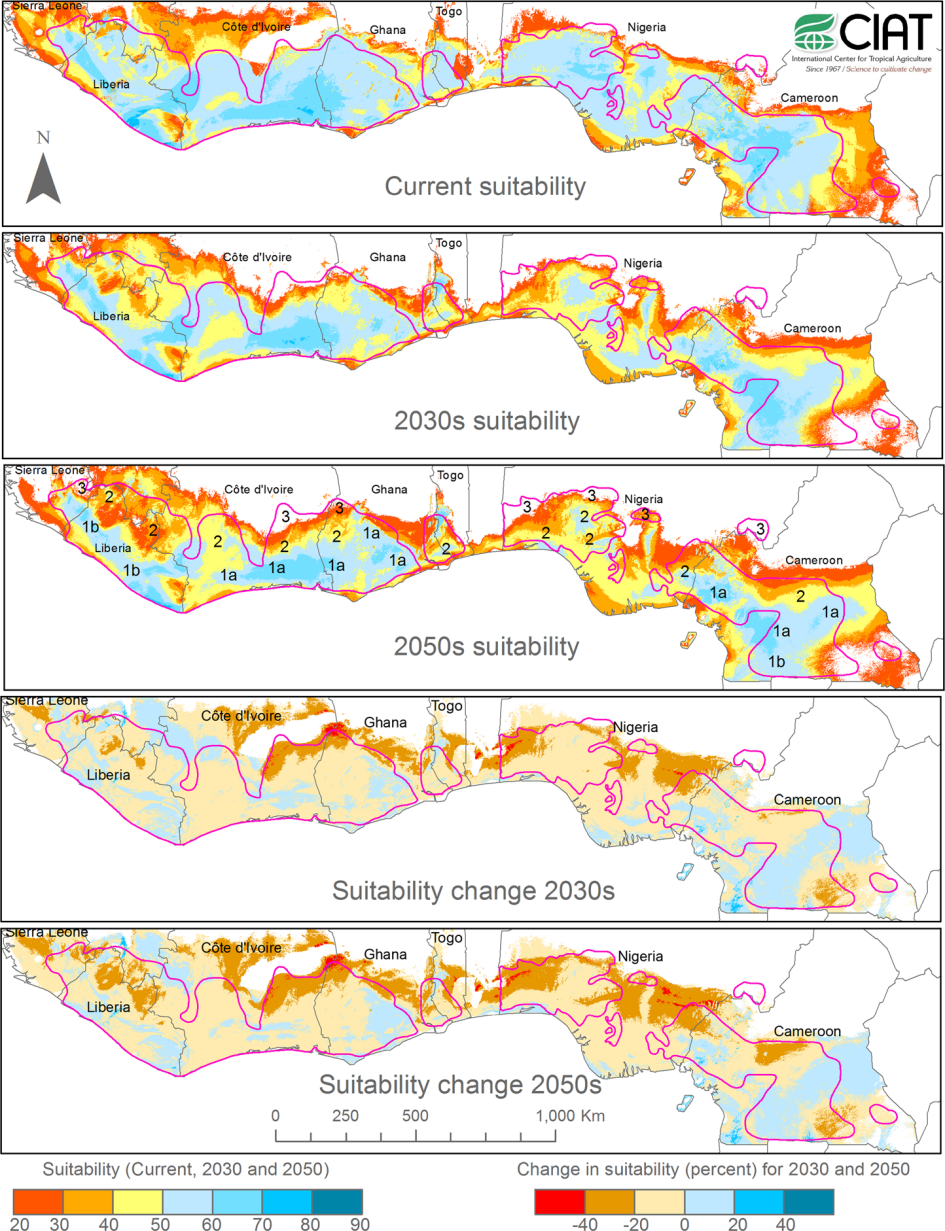
Relative climatic suitability (in percent) for cocoa (*Theobroma cacao*) as modeled with Maxent for the current and projected 2030s and 2050s climate in the West African cocoa belt, and corresponding suitability changes relative to the current climate. The red lines delimit current cocoa production areas. The *numbers* in the 2050s suitability map refer to the adaptation zones as defined in Table 1. Projected future climates are averages of 15 (2030s) and 19 (2050s) global circulation models

Based on the time sequence of the most likely scenario, we then assigned to the different parts of the cocoa belt one of three adaptation zones (one of these further subdivided into sub-zones) as defined in Table 1. Criteria for the zoning were (i) the current and projected 2050s climatic suitability of an area, whereby 50 % relative suitability according to the Maxent model was considered the lower threshold of “medium to high” suitability because most current cocoa producing areas have relative suitabilities above 50 % in the current climate (Fig. 1); and (ii) the projected trend in suitability (decrease or increase) between the current and projected 2050s climate. We do not propose clear limits between adjacent zones because spatial transitions of climate, and climatic suitability, in a topographically relatively flat region like West Africa are gradual without any clear cut-off values and “lines on the map” could be misunderstood as reflecting a deterministic approach to adaptation that would be contrary to our intentions. We also do not propose a cut-off value for unsuitable climates, which would be arbitrary, although we generally exclude areas with relative suitability below 20 % from our suitability maps. However, as an indication of the relative size of the areas belonging to the three suitability zones for the 2050s climate, we divide the total cocoa area into areas where the projected 2050s suitability is >50 % (highly suitable, Zone 1), between 20 and 50 % (intermediate suitability, Zone 2), and <20 % (unsuitable, Zone 3) and calculate the respective areas both in hectare and in percent of the total current cocoa area. The southeast of Cameroon was excluded from these calculations since relatively low suitabilities in this area may reflect the transition from the West African climate to that of the Congo basin and not be fully reliable.

**Table 1:** Schematic zoning of the West African cocoa belt according to the vulnerability of cocoa farming to climate change, focus of adaptation measures, and key requirements for their implementation per zone^a^

Zone	Current climatic suitability	Projected future climatic suitability	Current prevalence of cocoa farming	Focus of adaptation strategy	Key requirements	Regional examples
1a—intensification zone	Medium to high (>50 %)	Medium to high (>50 %)	(Co-)dominant crop in local farming systems	Sustainable intensification for increased yields and farmer income; diversification to buffer against market and environmental risks other than climate change	Technical assistance; input supplies including high-quality germplasm, fertilizer, pesticides (chemical or organic); affordable credit	Southern part of cocoa belts of Ghana and Côte d’Ivoire; parts of southern Cameroon
1b–expansion zone	Medium to high (>50 %)	Medium to high (>50 %)	Present but not dominant in local farming systems	Controlled expansion on existing agricultural and fallow land combined with forest conservation	Governance and monitoring systems ensuring land use planning and resource conservation; functioning supply chains for inputs and products; technical assistance; affordable credit	Southwestern part of Liberia with the exception of excessively humid coastal areas; parts of southern Cameroon
2–—diversification zone	Medium to high (>50 %)	Low to medium (20 to 50 %)	(Co-)dominant crop in local farming systems	Diversification of farming systems and supply chains with more heat and drought resistant crops; farm and landscape management for increased microclimatic protection (including shade use)	Functioning supply chains for a range of products including their inputs; legislation and administrative procedures encouraging farm trees; technical assistance; affordable credit	Northern parts of the cocoa belts of Liberia, Côte d’Ivoire, Ghana and Cameroon; most of cocoa area of Nigeria, Togo and Guinea
3—conversion zone	Low to medium (mostly 20 to 50 %)	Very low (<20 %)	Variable, not dominant in local farming systems	Diversification as a step in the progressive transition to alternative crops and supply chains that are better adapted to future climate conditions	Functioning supply chains for alternative crops and their inputs; technical assistance; affordable credit	Northeastern part of the cocoa belt of Côte d’Ivoire; northern and northwestern parts of cocoa belt of Nigeria; northernmost parts of cocoa belts of Ghana and Sierra Leone

^a^Percent suitability values refer to relative climatic suitability according to the Maxent model. For indicative location of adaptation zones see Fig. 1

For each of the zones, a set of adaptation actions was developed on the basis of literature information and the authors’ own field experience in this and other cocoa producing regions. As discussed further below, the results are intended as inputs into a participatory process involving local stakeholders, notably the farmers themselves as well as supply chain actors, to discuss and identify on-the-ground actions, and should not be understood as prescriptive. The emphasis and main objective of the paper is to illustrate a regional (as opposed to site-level) approach to climate change adaptation for a major commodity, rather than to propose detailed, farm-level adaptation actions.

## Results and discussion

### Variability of model scenarios

On average for the whole West African cocoa belt, an annual mean temperature of 25.5 °C now was projected to increase by 1.1 °C by the 2030s and 1.6 °C until mid-century (Table 2). Variation among global circulation models (GCMs) was considerable resulting in a difference of 0.8 °C between the lowest and highest quartile of model projections by the 2030s and 1.0 °C by the 2050s. Eco-physiologically more important is the maximum temperature of the warmest month (Läderach et al. 2013; Schroth et al. 2015b), falling into the dry season, which was projected to increase on average from 32.7 °C now to 33.8 °C in the 2030s and 34.2 °C in the 2050s. For the latter time period the variation between the lowest and highest quartile of the GCM projections was 1.6 °C (Table 2).

**Table 2:** Projected changes of key climate variables between current, projected 2030s and 2050s climates in the West African cocoa belt^a^

Variable	Current climate	2030s climate (15 GCMs)			2050s climate (19 GCMs)		
	Average	Average	Lowest quartile	Highest quartile	Average	Lowest quartile	Highest quartile
Annual mean temperature (°C)	25.5	26.6	26.2	27.0	27.1	26.6	27.6
Maximum temperature of warmest month (°C)	32.7	33.8	33.0	34.3	34.2	33.4	35.0
Annual precipitation (mm)	1809	1843	1728	1949	1857	1724	2000
Precipitation of driest month (mm)	20	20	14	25	21	14	28
Precipitation of driest quarter (mm)	100	99	84	114	101	82	122
Annual evapotranspiration (ETP, mm)	822	830	800	848	838	808	857
ETP of driest month (mm)	76	76	73	79	76	72	79
ETP of driest quarter (mm)	223	224	215	231	225	215	232
Excess precipitation over ETP for the driest month (mm)	−56	−56	−59	−54	−55	−58	−51
Excess precipitation over ETP for the driest quarter (mm)	−122	−127	−145	−109	−127	−144	−110
Consecutive months with <100 mm precipitation	3.9	3.0	2.7	3.2	2.8	2.7	3.2

^a^2030s and 2050s climate projections are according to 15 and 19 Global Circulation Models (GCMs), respectively. Variability among GCM projections are shown as the lowest and highest quartile of the distribution of projected climate values for the GCMs. All values are spatial averages for the entire cocoa belt

Annual precipitation was projected to increase slightly, with projections ranging from a slight decrease to a slight increase (Table 2). For the precipitation of the driest quarter, average projections showed almost no change by mid-century; however, projections ranged from a decrease from currently 100 to 82 mm for the lowest quartile to an increase to 122 mm for the highest quartile. At the same time, annual evapotranspiration (ETP) was projected to increase as a result of the increasing temperatures (Läderach et al. 2013), resulting in a slightly increasing water deficit during the driest quarter from 122 mm now to 127 mm in the 2050s. The variation among GCMs was again substantial, ranging from an increase of the deficit to 144 mm to a decrease of the deficit to 110 mm (Table 2). In contrast to this uncertainty about the evolution of dry season intensity, there was agreement among GCMs with regard to the length of the dry season (measured as consecutive months with less than 100 mm of rainfall), which all GCMs projected to become shorter. This relative uncertainty of climate projections for West Africa especially for rainfall has been highlighted by several authors (e.g., Niang et al. 2014). The relatively modest projected changes in rainfall in combination with the expectation of an overall shortening of the dry season has been interpreted in the sense that climatic suitability for cocoa will increasingly be influenced by maximum dry season temperatures, especially in the transition zones to the savanna, while seasonal drought will remain a threat (Läderach et al. 2013; Schroth et al. 2015b).

**Fig. 2:**
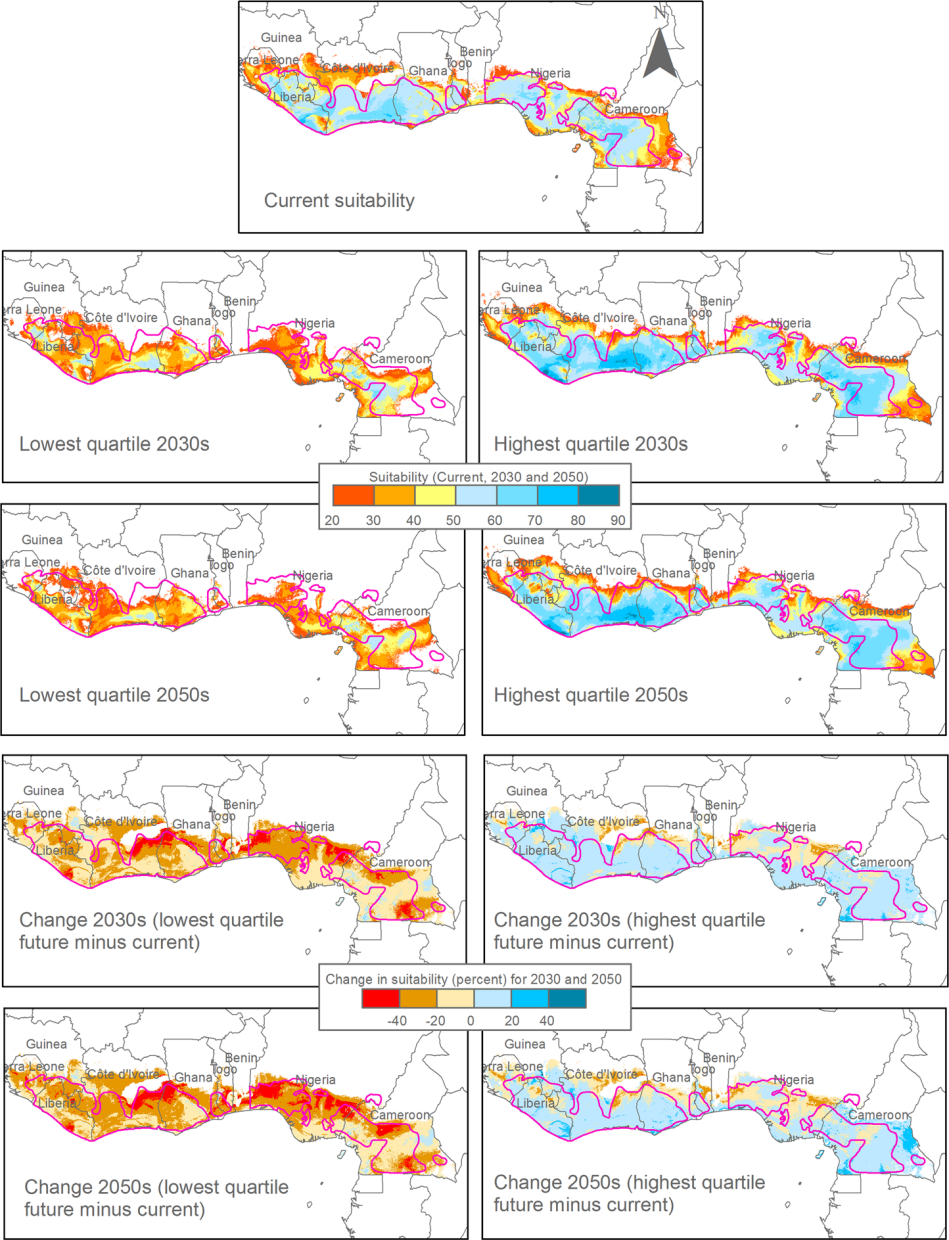
First and third quartile of relative climatic suitability projections (in percent) for cocoa (*Theobroma cacao*) of 15 (2030s) and 19 (2050s) global circulation models as modeled with Maxent for the West African cocoa belt, indicative of pessimistic (1st quartile) and optimistic (3rd quartile) climate change scenarios, and corresponding suitability changes relative to the current climate. The red lines delimit current cocoa production areas. The Maxent model of relative climatic suitability is the same as in Fig. 1

The variation of projections of individual climate variables as shown in Table 2 resulted in a correspondingly wide range of relative climatic suitability projections by the Maxent model for the two time horizons considered (Figs. 1 and 2). We discuss first the relative climatic suitability distribution as obtained by using as input for the future climate the average of all GCMs, corresponding to the most likely scenario if a “one model, one vote” rule applies (Tebaldi and Knutti 2010). We then compare this to the relative climatic suitability distributions obtained by using the highest and lowest quartiles of the GCM projections as proxies for the range of reasonably likely projections.

Our average model projected overall a modest decline in climatic suitability of the cocoa belt, with moderate decreases in relative suitability of 0 to −20 % in most areas for both time horizons (Fig. 1). Stronger decreases of −20 to −40 % were projected for the relatively hot and wet northeastern Liberia, where most of that country’s incipient cocoa sector is now located, and the hot and dry areas near the forest-savanna transition of northeastern Côte d’Ivoire and Nigeria. Smaller areas with significant suitability decline are in Togo and the northern edge of the cocoa belt of Cameroon. The progressive decrease in climatic suitability in these areas resulted in increasing areas in the northeast of the Ivorian cocoa belt and the northwest of the Nigerian cocoa belt falling below 20 % relative suitability. For comparison, relative suitability levels in the cocoa belt in the current climate are mostly above 50 % (Fig. 1). These areas were thus projected to become effectively unsuitable for cocoa by the 2030s. A small area in Sierra Leone was projected to become unsuitable by the 2030s, expanding further by the 2050s. On the other hand, in this average scenario there were large areas where projected climatic suitability remained medium to high (>50 %) and locally even increased by mid-century. These included some of the world’s most important cocoa producing areas in southern Côte d’Ivoire and Ghana, southern and southwestern Cameroon, and a large strip of land reaching from southern Liberia into Sierra Leone where currently only small amounts of cocoa are grown (Fig. 1).

This average scenario differed substantially from the pessimistic scenario represented by the 1st quartile of the GCM projections (Fig. 2). This scenario showed a drastic decline of projected climatic suitability for the cocoa belt as a whole, with decreases of relative suitability of >20 percent and even >40 percent being common. The most pronounced decreases were again in the northeast of the Ivorian and northwest of the Nigerian cocoa belt where large areas of land would become unsuitable for cocoa already by the 2030s, with the unsuitable area increasing further by the 2050s. However, the decline in relative climatic suitability would be almost universal throughout the cocoa belt. By the 2030s, areas with medium to high suitability (>50 percent) would be restricted to core areas of the Ivorian, Ghanaian and Cameroonian cocoa belts, declining further in size and suitability by the 2050s. On the other hand, the optimistic scenario of the 3rd quartile of the GCMs (Fig. 2) projected an improvement of climatic suitability by 0 to 20 percent in most of the West African cocoa belt with the main exceptions of the northeastern Ivorian and northwestern Nigerian cocoa areas as well as northeastern Liberia. With these exceptions, climatic suitability would be high to very high almost everywhere in the West African cocoa belt into the 2050s (Fig. 2).

### Scenario selection for adaptation zoning

How can such a wide range of suitability projections be converted into a strategy to reduce the vulnerability of the region’s cocoa producers and supply chains to climate change? We consider three options for this: (i) an all encompassing strategy that would cover all reasonably likely climate change scenarios as represented by the 1st and 3rd quartiles of the suitability models; (ii) a safety first strategy focusing on the pessimistic 1st quartile; and (iii) a “most likely scenario” strategy focusing on the average of all GCMs.

For the West African cocoa belt, the objective of the “all-encompassing” strategy seems impossible to achieve in view of the wide range of suitability projections. The more pessimistic projections of the 1st quartile would imply the need for a progressive disengagement from cocoa in most of the region with the exception of small parts of Cameroon and Côte d’Ivoire over the next roughly two decades and its replacement with more heat and drought adapted crops. The more optimistic projections (3rd quartile), on the other hand, would suggest no need for adaptation actions except in limited areas at the forest-savanna transition in Côte d’Ivoire, Nigeria and Sierra Leone. These two scenarios are obviously incompatible. A logical reaction to this discrepancy in suitability projections could be to delay adaptation decisions until climate models have improved and show less variation. However, in the meantime opportunities for adaptation may be missed, especially in the most affected and vulnerable areas, but also in those less affected areas where long-term investments in the productivity and sustainability of the cocoa sector are needed and justified.

The alternative, “safety-first” approach to adaptation would be to build an adaptation strategy on the pessimistic scenario of the 1st quartile projections to be on the safe side of possible future climate developments, as proposed in a different context by Lamichhane et al. (2015). However, while safeguarding against future surprises, this would imply major changes in the region’s farming practices and economies with significant costs and implications for livelihoods, despite the considerable areas projected under the most likely, average scenario to maintain a suitable climate for cocoa into the 2050s (Fig. 1). Such a pessimistic strategy could even have elements of a self-fulfilling prophecy by triggering a premature disengagement of public and private actors from the region’s cocoa sector. It might thus end up reinforcing the negative impacts of climate change on the region’s farming communities and economies.

In this situation of relatively high uncertainty but also considerable urgency, the most feasible approach for developing a regional climate change adaptation strategy is thus to follow the most likely, average scenario (Fig. 1) while keeping in mind the uncertainty of the models. The strategy should then be updated every few years as new data become available and climate models become more refined and regionally adapted. This is the approach we are taking now.

### Adaptation zoning of the West African cocoa belt

Our model of current climatic suitability for cocoa farming and projected average climatic suitabilities for the 2030s and 2050s allows to divide the West African cocoa belt into three broad zones of increasing vulnerability to climate change (Table 1). These zones have no clear-cut boundaries that could be shown on a map, but transition gradually into each other (Fig. 1).

Zone 1 includes those areas where relative climatic suitability for cocoa farming is medium to high now and projected to remain so into the 2050s. The projected changes in suitability are small and can range from weakly negative to positive. This zone comprises an arc of land along the Guinea coast from Sierra Leone through southwestern Liberia and southern Côte d’Ivoire to eastern Ghana, and a second, smaller arc covering much of southern Cameroon (Fig. 1). It excludes the driest and hottest parts of the forest belt closest to the savanna, but also some very wet coastal areas where cocoa would suffer from high fungal disease pressure (Wood and Lass 2001). It includes some of the world’s foremost cocoa production areas, such as the Western Region of Ghana and the south of Côte d’Ivoire and Cameroon (Zone 1a), but also areas where cocoa is currently present only as a minor crop in a still widely forested environment, such as southwestern Liberia (Zone 1b).

Zone 2 is a transition zone that includes those areas of the current cocoa belt that are intermediate between Zones 1 and 3. Generally, Zone 2 areas have a negative trajectory of projected climatic suitability, although the trend is sufficiently modest and/or current climatic suitability sufficiently high for these areas to remain broadly suitable for cocoa farming into the 2050s. Zone 2 comprises a mosaic of situations ranging from small and decreasing patches of land where climatic suitability for cocoa is projected to remain high (such as the highlands of Togo or a small core area of the cocoa belt of western Nigeria), to areas where suitability is projected to decline without reaching the very low levels of Zone 3. Overall, Zone 2 areas show a clear regression of projected climatic suitability among the three successive time steps, as can for example be seen in western Côte d’Ivoire and northeastern Liberia (Fig. 1).

Zone 3, finally, includes those areas where over the next few decades climatic suitability is projected to fall to such low levels that their continued ability to support cocoa farming is highly questionable. These are generally areas near the forest-savanna transition that already experience the lowest climatic suitability levels of the whole cocoa belt now because of their long dry season and high drought risk. In these areas, climatic suitability is projected to decline most drastically as maximum dry season temperatures approach the physiological limits of cocoa (Läderach et al. 2013; Schroth et al. 2015b). Zone 3 areas are concentrated in northeastern Côte d’Ivoire and a small adjacent part of Ghana, the northern and western fringes of the cocoa belt of Nigeria, and the northeastern extremes of the cocoa belt of Sierra Leone (Fig. 1).

To give an idea of the relative size of the areas of various suitability levels, by the 2050s areas with climatic suitability >50 % (roughly corresponding to Zone 1) would cover 22.4 million hectares or 38 % of the current cocoa belt, areas with climatic suitability between 20 % and 50 % (Zone 2) would cover 32.2 million hectares or 55 % of the current cocoa belt, and areas with climatic suitability <20 % (Zone 3) would cover 4.1 million hectares, or 7 % of the current cocoa belt.

### Crop development and adaptation strategies per zone

Table 1 summarizes the main characteristics and options for cocoa development and adaptation for each zone. The most positive scenario applies to adaptation Zones 1a and 1b, dubbed respectively as the intensification and expansion zones (Table 1). While these sub-zones share favorable climate projections for mid-century, they differ in their current prevalence of cocoa farming.

Zone 1a (“intensification zone”) includes areas with a strong historical and current role of cocoa in the farming landscapes and local economies, while in Zone 1b (“expansion zone”) cocoa is currently not the dominant crop. Conditions in Zone 1a are thus conducive to investments in the intensification of cocoa farming to sustainably increase per-hectare yields and farmer incomes from currently low-average levels. This includes the renovation and replanting of old farms and improved farm management such as nutrient, pest and disease management to increase yields. Opportunities to expand cocoa farming to new land are rare in this zone which is largely saturated with cocoa to the point of cocoa farms having widely encroached on protected areas (Bitty et al. 2015). Since investments in farm replanting and the rehabilitation of soil fertility show results mostly after several years, it is important for farmers and supply chain actors to know that climatic conditions in this zone are projected to remain favorable for the next one or two generations of farmers and cocoa trees. This is also the zone where investments in infrastructure and supply chains (e.g., road improvement, cocoa buying stations, networks and infrastructure for supplying inputs such as seeds and fertilizer) are subject to the lowest climate risks and may therefore be most attractive to private sector investors or the use of public loan funds.

Zone 1b (“expansion zone”), in contrast, includes areas where cocoa farming could increase from a climatic point of view, thereby compensating for future production losses in more negatively affected areas. However, this expansion involves an obvious risk of undesirable environmental impacts through deforestation, especially where it concerns areas that have still considerable forest reserves such as Liberia and Cameroon. The main challenge in this sub-zone is thus to coordinate agricultural and forest policies to ensure that farm expansion occurs under zero-deforestation principles on already deforested land (Brown and Zarin 2013; Schroth et al. 2016). This includes the risk that a high-value crop like cocoa, if expanding on existing agricultural and fallow land, could displace current lower-value land uses such as slash-and-burn farming into forest areas, thereby causing indirect deforestation. On the other hand, a high-value but labor-intensive crop like cocoa could help improve the livelihoods of current residents on a smaller area of land compared to land uses that generate less income per unit area, including slash-and-burn agriculture. The deforestation risk is particularly high if production expansion involves substantial human migrations, as has often been the case in the history of cocoa farming in West Africa and elsewhere (Ruf et al. 2015). Zone 1b thus combines significant economic opportunities and environmental risks and therefore requires strong governance frameworks and independent monitoring in developing these opportunities. It is also the area where a long-term view on climatic suitability (2050s and beyond) is particularly important to avoid setting up new cocoa farms and supply chains in areas that may lose their climatic suitability within the near future.

Zone 2 is dubbed diversification zone in Table 1 in reference to one key component of an adaptation strategy where decreasing climatic suitability for cocoa implies an increasing risk of crop failure, especially during extreme years (Altieri et al. 2015). Tree crop farmers in West Africa have often responded to market and environmental pressures with diversification of their farming systems, including in areas where farming systems have come under pressure from pests, diseases, weeds and degraded soil and microclimatic conditions (Schroth and Ruf 2014; Ruf and Schroth 2015). This process can be a step in a progressive shift to an alternative main crop, but often it leads to a greater diversity of farming systems within the landscape. Common examples from West Africa include cocoa farmers adopting rubber (*Hevea brasiliensis*), oil palm (*Elaeis guineensis*), citrus (*Citrus* spp.), or cashew (*Anacardium occidentale*) as additional or alternative crops. Crop diversification is a particularly important strategy under conditions of declining climatic suitability for the main crop, provided that crops are added that are better adapted to the hotter future climate of the region (Lin 2011; Jalloh et al. 2013). While a degree of diversification as a strategy to reduce market and environmental risks is also advisable in Zone 1, it is less urgent there than in Zone 2. Diversification not only implies changes in farming practices, but needs to involve the whole supply chains and thus the private sector, especially where crops are added to farming systems that have no well-developed local markets yet. Since in Zone 2 cocoa is likely to remain a key component of farming systems over the coming decades despite increasing environmental pressures, it is also here that the adaptation of cocoa farming practices to the changing climatic conditions is particularly important. So far, discussions about the adaptation of cocoa to climate risks in West Africa have focused mostly on water (Carr and Lockwood 2011), but increasing maximum temperatures during the dry season are also projected to become main stressors of cocoa trees in West Africa (Läderach et al. 2013; Schroth et al. 2015b). The only practical way of reducing heat stress in tree crops like cocoa is through shading by appropriately selected and spaced companion trees (Lin 2007). Cocoa—an understory crop of the Amazon forest—has traditionally been grown under the shade of forest remnant trees or planted shade trees, but over the last decades, shade use in West Africa has tended to decrease (Ruf 2011; Ruf and Schroth 2004). Increasing the use of shade in cocoa farms against this trend is thus particularly important as an adaptation measure in this zone.

Zone 3, finally, is termed the “conversion zone” in Table 1 because here climatic conditions are projected to become progressively unsuitable for cocoa, as indicated by projected very low relative climatic suitability levels (Fig. 1). In this zone, increasing maximum dry season temperatures approaching the physiological tolerance of the crop will compound the already high risk of seasonal drought (Läderach et al. 2013; Ruf et al. 2015; Schroth et al. 2015b). Unless cocoa varieties with significantly higher tolerance to heat and drought will become available over the next decade or so, it is likely that Zone 3 areas will cease to produce cocoa within the near future. Even if such new varieties become available, they may not be introduced or not at the necessary scale in this most risky of all cocoa production environments. To avoid that this leads to economic hardships at the local level, the transition to alternative crops and farming systems needs to be facilitated through suitable adaptation measures. This will most likely involve again an initial step of diversification of farming systems with more drought and heat tolerant crops (with cashew—*Anacardium occidentale*—being one example among others), possibly through subsidized credit packages supported by the corresponding marketing structures and extension services. Differently from Zone 2, however, there would be a clear expectation here for diversification to be only a step in complete crop change and the progressive abandoning of cocoa, although this should happen progressively at the farmers’ own pace. New cocoa planting in the zone may thus not be encouraged or subsidized, but should also not be prevented, keeping in mind that West African farmers have occasionally planted cocoa with success on savanna land that agronomists would have considered unsuitable for the crop (Jagoret et al. 2012). Recommendations to increase the use of shade trees in cocoa farms are less indicated in this zone than in Zone 2. A dense shade canopy may reduce the farmers’ flexibility to adapt their farming systems through incorporation of new, presumably less shade-tolerant crops than cocoa and may compete with cocoa for water during the dry season (Willey 1975). On the other hand, tree planting on farm boundaries, along roads and watercourses and in the wider landscape would be helpful to maintain favorable microclimatic conditions and protect the crops from increasingly hot dry season winds (Brenner 1996; Nuberg and Bennell 2009).

### Time horizon and boundaries

While the proposed adaptation zones and corresponding adaptation measures are thus reasonably well-defined, any boundaries between these zones would be arbitrary since climate and climate change do not show hard boundaries and projections are dependent on the time horizon chosen. This is especially the case for Zone 2 where a progressive loss of climatic suitability is evident between the 2030s and the 2050s (Fig. 1). In Fig. 1, we indicated the three zones for the projected situation of the 2050s emphasizing a longer-term view of vulnerability and adaptation needs and recognizing the considerable time that comprehensive adaptation measures may take to implement on such a large scale and across several countries. We do, however, recognize that the most appropriate time frame for adaptation planning is situation dependent. For example, a shorter time horizon would be more appropriate for adaptation decisions in current cocoa producing areas (Zones 1a and 2; Table 1) and a longer time horizon more appropriate when expansion into new areas is considered (Zone 1b). Also, recommendations for straight-forward changes in farming practices such as shading or pest and disease control (e.g., Zone 2) may be based on short-term projections while decisions about the breeding of new tree crop varieties or the progressive shift to alternative crops (Zone 3) would require a longer planning horizon. Finally, governments designing rural development strategies may take a longer-term perspective of climatic suitability than private sector actors deciding about supply chain investments in a given locality. In many cases, concrete adaptation actions may be planned for a 2030s time horizon, but considering the longer-term climate prospects of the area and periodically updating adaptation plans as new information becomes available. This approach also takes into account the uncertainty about future emissions scenarios (Moss et al. 2010), which will determine whether a certain projected change in climate will occur some years earlier or later.

The arbitrariness of any fixed boundaries between zones precludes a deterministic approach to adaptation planning where certain measures were restricted to (or even made compulsory in) one zone and precluded from neighboring ones, with the zone boundary acting as a hard limit to funding and technical assistance programs. For example, farmers in Zone 2 wishing to intensify their cocoa systems should be supported in doing so, including through technical recommendations appropriate for their specific geographic location. This may include a greater emphasis on useful trees for shade and diversification compared to farmers pursuing farm intensification in a Zone 1 location. Similarly, farmers in Zone 3 willing to replant their farms with cocoa rather than an alternative crop should not be discouraged, but rather be advised about the projected climatic trajectory of the location and helped with the identification of suitable crops for the diversification of their farming systems. Farmers in transition areas between zones should be aware of recommendations on both sides and be free to choose or assemble the mix of options they find most suitable for their situation.

In practical terms, we suggest that zoning should be used as an input into a participatory process of stakeholder engagement at various scales, from the local to the national and regional scale. At the local scale, informed and sensitized farmers can make better choices about their crops and farming practices (Pettengell 2010), and local government and civil society bodies as well as private companies can then support farmer preferences through technical assistance, credit and input supplies matching the local demand. The pre-defined adaptation zones then progressively take further shape and are refined through the spatial patterns of informed, local demand. In this way, impact-based (“top down”) and capacity-based (“bottom up”) approaches to climate change adaptation^1^ are integrated in the process of defining concrete, spatially explicit adaptation plans and actions (Vermeulen et al. 2013). At larger scales, the zoning can help inform governments and supply chain actors about investment opportunities and needs (e.g., in sustainable intensification or the setting up of new supply chains in areas of diversification and progressive crop change) and could help project and monitor the future supply of cocoa from the region. It could help communicate to various audiences that climate change is not a process of uniformly negative impact, but one that is re-creating and reshuffling a patchwork of situations where production increase in some areas could at least partly compensate for production decline in others.

### Intensity of change: types and levels of adaptation

Our adaptation zoning approach implies that changes in farming practices of various types and intensities are needed depending on the location in the West African cocoa belt, and also the time frame chosen. Moreover, these changes could be planned and facilitated at a local scale through local institutions or integrated at larger scales and through higher-level institutions. In attempts to characterize the intensity and complexity of change involved in reducing the vulnerability of local people to climate change, various typologies of climate change adaptation have been proposed. These include the distinction of Incremental, Systemic and Transformational adaptation proposed by Vermeulen et al. (2013) and used by the Climate Change and Food Security (CCAFS) program of the CGIAR, focusing mostly at the technical level and the farm scale. The similarly three-level system of Resilience, Transition and Transformation used by Pelling (2011), on the other hand, emphasizes social changes, ranging from relatively simple measures to increase the resilience of communities to climate shocks to societal changes addressing the root causes of their vulnerability.

In Table 3 we draw on both systems to illustrate the various intensities and levels of change involved in a comprehensive, regional approach to climate change adaptation for the West African cocoa belt. At the farm scale, this would range from better farm management and modest changes in farming practices without affecting the role of cocoa as the principal crop (especially Zone 1a), through the diversification of farms and livelihoods to reduce the dependence on cocoa (especially Zone 2), to the progressive change to alternative crops that are better adapted to future climatic conditions (Zone 3). These changes correlate with our three adaptation zones, with the caveats discussed earlier that measures particularly emphasized in one zone may also be relevant, if less urgent, in others (e.g., various degrees of diversification and intensification may take place in all zones). It is also important to recognize that at this scale, the three levels of change do not necessarily imply increasing levels of difficulty in adopting or facilitating the corresponding changes, as the terminology may suggest. As mentioned earlier, the diversification of farming systems (a systemic change) through adoption of additional tree crops, sometimes leading to transformative crop change, is a common reaction among cocoa farmers in West Africa to mounting environmental pressures and market risks (Ruf and Schroth 2015). On the other hand, the success of efforts to (incrementally) intensify West African cocoa production systems through replanting of old farms, better tree husbandry and increased inputs of agrochemicals has so far been relatively modest. It appears, then, that at least in the West African cocoa world, systemic and transformative changes could sometimes be easier to achieve than incremental change.

**Table 3:** Examples of interventions illustrating the three types or intensities of adaptation at three intervention levels (farm or technical, government, and private sector level)^a^

Intervention level	Type of adaptation		
	Incremental adaptation (resilience)^b^	Systemic adaptation (transition)	Transformational adaptation (transformation)
Technical, farm level	• Better planting material (all zones) • Increased shade use (especially Zone 2) • Standard agronomic practices to increase profitability (fertilizer, pruning, grafting) (especially Zones 1 and 2)	• Diversification of farms and livelihoods (all zones, but especially Zones 2 and 3)	• Progressive change to alternative crops that are more adapted to future environmental conditions (Zone 3)
Governments, international donors and development agencies	• Time-limited and site-level adaptation projects focusing on most critically affected areas • Input subsidies (e.g., fertilizer, crop and shade tree seedlings)	• Open-ended adaptation programs covering entire national production area with site specific measures • Planned farm diversification programs depending on area • Universal access to technical assistance • Locally differentiated finance programs to help farmers adapt (e.g., transition to other crops, increase shade, replant, intensify) • Legal changes incentivizing native tree planting and retention on farms	• Coordinated regional adaptation planning • Integration with forestry and conservation policies, zero-deforestation policies • South-south collaboration among countries to exchange germplasm and information, assist controlled expansion in suitable areas, and prevent uncontrolled production expansion into unsuitable areas and/or negatively affecting prices and environment
Private sector	(same as for Government)	• Adaptation along single supply chains	• Coordinated adaptation across multiple supply chains of alternative crops to facilitate crop diversification and change

^a^The terminology for the type of adaptation follows Vermeulen et al. (2013) and Pelling (2011), in brackets

^b^The term “coping” is also sometimes used for this type of adaptation, but other authors contrast coping as a short-term strategy to deal with natural climate variation with adaptation as a long-term strategy to deal with changing environmental conditions and increased risk; see Pelling (2011) and Füssel (2007)

At organizational levels above the farm, the three types of adaptation can take on additional meanings, reflecting a change from site-level interventions and technical recommendations to supportive systems at increasing spatial scales. Currently, climate change adaptation in agriculture is mostly characterized by incremental measures and projects, attempting to locally increase the resilience of farming communities to climate shocks, including by increased access to inputs to increase productivity. Such initiatives are usually confined to certain project sites and their beginning and end defined by funding cycles (Table 3). A more comprehensive, systemic approach to adaptation would make it an integral, open-ended part of national agricultural development policies supported by universal access to competent and site specific technical assistance, locally differentiated finance programs and legal-administrative changes (such as legislation incentivizing the planting of trees on farms). At a still more comprehensive, transformational level, adaptation planning could be scaled up and integrated at the regional level through south-south cooperation among governments and supply chain actors. At this level, the transfer of technology and the coordination of actions among countries could be organized, for example to prevent sudden declines or increases of cocoa supply that could be disruptive on the market, or to channel the establishment of new cocoa farms to the areas with the highest long-term climatic suitability and least risk of deforestation. Finally, the private sector could adopt roles of increasing complexity within this regional adaptation framework, from supporting local projects to adapting entire value chains of individual crops (Nyasimi et al. 2014) to adapting and coordinating the value chains of various crops to the challenges resulting from large-scale diversification and crop change (Table 3).

While we develop and illustrate this regional approach to climate change adaptation for cocoa in West Africa, it is by no means specific to the crop or the region. As mentioned earlier, local declines of the climatic suitability for Arabica coffee have been predicted for parts of Mesoamerica and Indonesia, with potentially severe impacts on the local economies and the supply of coffee from the affected regions. Through regional planning and integration of adaptation responses, such local declines in production could partly be mitigated through planned intensification and expansion elsewhere. This is especially the case where parts of a larger producing region are projected to be less or differently affected by climate change than others, as has been shown for Arabica coffee production across the Indonesian archipelago (Schroth et al. 2015a) and Mesoamerica (Ovalle-Rivera et al. 2015). Small-scale variability in climatic vulnerability within these countries can also be high (Baca et al. 2014; Schroth et al. 2015a), suggesting that through careful zoning followed by investments in appropriate adaptation actions per zone, negative economic impacts on countries and regions that are highly dependent on a single, climate sensitive crop species might be reduced or avoided.

## Conclusions

Climate change adaptation is often conceived and implemented as a process intended to cut losses and reduce hardships, focusing typically on areas where local communities are already under strong climatic pressure and at a relatively local scale. In contrast to climate change mitigation, which is generally seen as a global activity, climate change adaptation is mostly considered a local process (Agrawal 2008; Pelling 2011). Differently from coffee, cocoa has so far not been the object of major efforts to reduce its vulnerability to climate change, although some studies have shown that for parts of the West African cocoa belt this would be necessary and other areas will soon follow (Läderach et al. 2013; Ruf et al. 2015; Schroth et al. 2015b). Rather than adopting a local approach, we propose here an approach to climate change adaptation for cocoa in West Africa that focuses at the regional level and that emphasizes the different degrees of vulnerability to climate change of various parts of the cocoa belt. We highlight not only those areas where the deterioration of climatic conditions will require the adaptation of farming systems (and crops) to new climatic conditions, but also those areas where the relative absence of climatic risks should encourage investments in the intensification and, possibly, expansion of cocoa production systems. By dividing the cocoa belt of West Africa into zones of vulnerability to climate change and identifying a set of actions appropriate for each zone, we go a step from a local, crisis management approach towards a regional, sustainable development approach to climate change adaptation. This can also be conceived as a change from incremental, site-level adaptation projects to transformative, regional adaptation planning. We emphasize that the identified zones are tentative and should serve as an input to a more local process of stakeholder engagement to identify the most suitable, demand-driven actions per site. Through this regional approach, losses in cocoa production in some areas could be compensated by gains in others, and investments in either cocoa or alternative crops could be channeled to the most appropriate locations. The region’s ability to supply the majority of the world’s cocoa could thus be maintained for the years and decades to come, even if farmers in some areas may need to shift to alternative crops while their peers in other zones intensify and increase production. The regional development approach to climate change requires for its implementation that adaptation planning be conducted and actions coordinated at least at a national (as opposed to site) level, but preferably be integrated across the region, despite the institutional challenges that this poses. While we focus here on cocoa in West Africa to illustrate the regional approach to climate change adaptation planning, this approach equally applies to other agricultural commodities, especially where these are of major regional importance for the economies of developing countries and the livelihoods of their inhabitants.

## Appendix

**Table 4:** List of variables used in the modeling of current and future climatic suitability for cocoa of the West African cocoa belt with Maxent

Variable	Variable definition	Current average
BIO 1	Annual mean temperature	25.5 °C
BIO 2	Mean diurnal range (mean of monthly (max temp–min temp))	9.7 °C
BIO 3	Isothermality (BIO 2/BIO 7) (*100)	74
BIO 4	Temperature seasonality (standard deviation *100)	94.6
BIO 5	Maximum temperature of warmest month	32.6 °C
BIO 6	Minimum temperature of coldest month	19.4 °C
BIO 7	Temperature annual range (BIO 5–BIO 6)	13.1 °C
BIO 8	Mean temperature of wettest quarter	25.0 °C
BIO 9	Mean temperature of driest quarter	25.8 °C
BIO 10	Mean temperature of warmest quarter	26.6 °C
BIO 11	Mean temperature of coldest quarter	24.2 °C
BIO 12	Annual precipitation	1814 mm
BIO 13	Precipitation of wettest month	315 mm
BIO 14	Precipitation of driest month	23 mm
BIO 15	Precipitation seasonality (coefficient of variation)	61
BIO 16	Precipitation of wettest quarter	772 mm
BIO 17	Precipitation of driest quarter	115 mm
BIO 18	Precipitation of warmest quarter	345 mm
Cons_mths	Consecutive dry months (<100 mm rainfall per month)	3.7
ETP 1	Annual evapotranspiration	816 mm
ETP 3	Evapotranspiration of driest month	75 mm
ETP 5	Evapotranspiration of driest quarter	221 mm
ETP 6	Evapotranspiration of warmest quarter	223 mm
ETP 8	Excess precipitation over evapotranspiration during driest quarter (BIO 17 - ETP 5)	−106 mm

**Table 5:** Global circulation models (GCMs) included in the modeling of future climatic suitability of cocoa (*Theobroma cacao*) in West Africa and their countries of origin. Data were obtained from http://www.ccafs-climate.org

Model	Country	Institute
bcc_csm1_1	China	Beijing Climate Center
bcc_csm1_1_m	China	Beijing Climate Center
ccsm4	USA	National Center for Atmospheric Research
cesm1_cam5	USA	National Center for Atmospheric Research
csiro_mk3_6_0	Australia	Commonwealth Scientific and Industrial Research Organisation
fio_esm	China	First Institute of Oceanography
gfdl_cm3	USA	National Oceanic and Atmospheric Administration
gfdl_esm2g	USA	National Oceanic and Atmospheric Administration
gfdl_esm2m	USA	National Oceanic and Atmospheric Administration
giss_e2_h	USA	National Aeronautics and Space Administration
giss_e2_r	USA	National Aeronautics and Space Administration
hadgem2_ao	UK	Hadley Center
hadgem2_es	UK	Hadley Center
ipsl_cm5a_lr	France	Institut Pierre-Simon Laplace
miroc5	Japan	University of Tokyo
miroc_esm	Japan	University of Tokyo
miroc_esm_chem	Japan	University of Tokyo
mri_cgcm3	Japan	Meteorological Research Institute
noresm1_m	Norway	Norwegian Climate Centre

**Table 6:** Variables making a contribution higher than 5 % to explaining variation in climatic suitability for cocoa in the West African cocoa belt according to a Maxent model based on 24 climate variables (see Appendix Table 4)

Variable code	Variable	Contribution to variation in percent (%)
BIO 14	Precipitation of driest month	28.8
BIO 4	Temperature seasonality	23.9
BIO 15	Precipitation seasonality	10.8
BIO 17	Precipitation of driest quarter	8.9
BIO 9	Mean temperature of driest quarter	6.5
BIO 2	Mean diurnal temperature range	6.0

## Footnotes

1 Füssel (2007) uses the terms hazard-based *versus* vulnerability-based approaches to climate change adaptation in a similar context.
